# A unique cytoskeleton-associated protein in *Cryptococcus neoformans*

**DOI:** 10.1080/21505594.2018.1451185

**Published:** 2018-04-24

**Authors:** Angie Gelli

**Affiliations:** 1 Department of Pharmacology, SOM, University of California, GBSF 3503, 451 Health Sciences Dr. Davis CA

**Keywords:** Cgp1, CAP-Gly domain, cytoskeleton, *Cryptococcus neoformans*, microtubules, cryptococcal meningoencephalitis, virulence

C*ryptococcus neoformans* (Cn) is the leading cause of fungal infections of the central nervous system and accounts for significant patient morbidity and mortality [1]. The patients most susceptible are those that are immunocompromised including, HIV patients, organ transplant recipients and those with immunosuppressive therapy or malignancy [2]. Approximately 223,100 cases and 181,100 annual fatalities of cryptococcal meningoencephalitis (CM) have been estimated to occur globally. Approximately 73% of all cases occur in sub-Saharan Africa [1]. CM also causes significant morbidity and mortality in the United States. Of 30,840 hospitalizations attributed to CM between 1997 and 2009, approximately 3,440 deaths were reported [3]. Of all the CM cases, 21.6% occurred among HIV-uninfected patients and although a steady decline in HIV-infected deaths has been documented, there appears to be a persistent burden of CM among HIV-uninfected patients [3]. Neurologic sequelae including visual loss, cranial palsies, neurologic deficit or mental impairment occurs in 40–50% of all treated patients and 20–25% experience a relapsing course [4]. Without rapid intervention, CM is universally fatal regardless of the immune status of the host. Collectively, these issues warrant significant studies that resolve the biology of *C. neoformans* and the mechanisms of pathogenicity.

In a recent compelling study, Wang et al., identified a unique cytoskeleton-associated protein and examined its role in the pathogenesis of *C. neoformans* [5]. All eukaryotes consist of cytoskeletal filaments that include microtubules made from tubulin. Among the cytoskeleton-associated proteins (CAPs) are those that contain a protein-interaction module that consist of a highly conserved CAP-glycine-rich (CAP-Gly) domain [6]. An evolutionary conserved feature of CAP-Gly proteins is their capacity to associate with tubulin monomers, dimers and /or microtubules via the C-terminal EEY/F sequence motifs in tubulin and in microtubule end-binding proteins [7]. The recruitment of CAP-Gly proteins to microtubules leads to the regulation of several cellular processes including the arrangement and dynamics of microtubules, intracellular signaling, chromosome segregation, cell migration and vesicle transport [6].

CAP-Gly proteins have been characterized in *Saccharomyces cerevisiae* and in *Saccharomyces pombe*, however the functional activity of CAP-Gly proteins in fungal pathogens are unknown thus underscoring the relevance of the study by Wang et al. The goal of the study was to identify CAP-Gly proteins in *Cryptococcus neoformans* and to establish whether CAP-Gly proteins mediate mechanisms of pathogenesis [5]. The authors identified five genes predicted to encode CAP-Gly domains and subsequently used an in silico approach to analyze the protein domain structure of each gene. Of the five genes identified (including, Pac2, Nip100, Alf1 and Bsp3) one in particular stood out. The CAP-Gly protein, CNAG_06352 in *C. neoformans* consisted of 1,057 amino acids with additional domains, namely SPEC and Spc7. This newly identified gene was given the name Cgp1 (CAP-Gly protein 1) and chosen for further characterization because of its unique domain structure.

Interestingly, the authors found that even though Cgp1 contained three distinct motifs, the CAP-Gly domain appeared to be solely responsible for the key roles of Cgp1. Deletion of the Spc7 domain did not alter microtubule-related functions and sexual differentiation and the SPEC domain appeared to play only a very minor role in microtubule stability and filamentous growth. On the other hand, the CAP-Gly domain appeared to be required for all the functions observed for Cgp1 in *C. neoformans* [5].

Based on previous characterizations of CAP-Gly proteins, it has been well established that the CAP-Gly domain associates with the EEY/F motifs in alpha-tubulin; however other proteins containing end-binding homology domains, zinc-finger motifs and proline-rich regions are also capable of recruiting CAP-Gly proteins. Collectively, these multiple partners suggests that CAP-Gly proteins likely function in different biological processes. In fact, the study by Wang et al., found that Cgp1 appeared to have pleotropic roles in the biology and pathogenesis of *C. neoformans* [5].

Wang et al., made a significant discovery. They showed very elegantly that one of the CAP-Gly proteins (Cgp1) in *C. neoformans* is not only required for microtubule-mediated cellular functions, but they also demonstrated that Cgp1 regulates virulence and differentiation of *C. neoformans* [5]. The attenuated virulence is likely due the defects in melanin synthesis, growth at high temperatures and general stress responses [8]. Considering the roles of Cgp1 in the different aspects of the pathobiology of *C. neoformans*, an attempt was made to resolve the Cgp1-mediated regulatory networks. To do this, Wang et. al., set out to identify binding partners of Cgp1 through in vitro pulldown assays and proteomic analysis [5]. The alpha- and beta-tubulins were among the proteins identified as interacting with Cgp1 and the use of CryptoNet revealed an in-depth Cgp1-linked regulatory network with ties to a diverse array of biological processes including stress response and adaptation, transport, cell cycle, and protein folding/catabolic activity. Collectively, the data presented by Wang et al., strongly suggest that Cgp1 maintains an integral role in different cellular processes that are central to the pathobiology of *C. neoformans*. Although a previous study performed a partial analysis of CNAG_06532 and concluded that CNAG_06532 functions as a transcription factor, Wang et al., reported several observations that do not support this claim [9]. Through their detailed and thoughtful study, the data presented by Wang et al., support that notion that Cgp1 (CNAG_06532) is a cytoskeleton-related protein with a CAP-Gly domain that functions within diverse aspects of *C. neoformans*’ biology.

Given the vast regulatory effects of Cgp1 in *C. neoformans*, the possibility that Cgp1 may be a viable target for the development of antifungal drugs must be considered. Although, the conserved activity of CAP-Gly proteins among eukaryotes may limit specificity for *C. neoformans*, if repurposed drugs, small molecules or peptides capable of blocking Cgp1 activity can function as antifungal adjuvants, then a combination of existing therapies with inhibitors of Cgp1 activity may provide additional treatment options for CM [10].
